# Depression in Parkinson’s disease is associated with reduced ventral striatal dopamine transporter binding

**DOI:** 10.1038/s41531-026-01363-2

**Published:** 2026-05-25

**Authors:** Jessica E. M. C. Dirkx, Filip Grill, Nienke Dijk, Roshan Cools, Jan Booij, Bastiaan R. Bloem, Rick C. G. Helmich, Henricus G. Ruhé, Maria H. C. T. van Beek

**Affiliations:** 1 Department of Psychiatry, Radboud University Medical Centre (Radboudumc), Nijmegen, the Netherlands; 2 Donders Institute for Brain, Cognition and Behaviour, Centre for Cognitive Neuroimaging, Radboud University Nijmegen, Nijmegen, the Netherlands; 3 Aging Research Center, Karolinska Institutet and Stockholm University, Stockholm, Sweden; 4 Department of Radiology and Nuclear Medicine, Amsterdam University Medical Centres, University of Amsterdam, Amsterdam, the Netherlands; 5 Department of Medical Imaging, Radboudumc, Nijmegen, the Netherlands; 6 Department of Neurology, Center of Expertise for Parkinson & Movement Disorders, Radboudumc, Nijmegen, the Netherlands

**Keywords:** Diseases, Neurology, Neuroscience

## Abstract

Major depressive disorder (MDD) is a common non-motor symptom in Parkinson’s disease (PD), affecting one third of patients. Dopaminergic deficits have been hypothesised to contribute to PD-MDD, but direct molecular evidence is limited. In this cross-sectional study, we compared striatal dopamine transporter (DAT) binding between 22 depressed (DSM-5) and 34 well-matched non-depressed PD patients using [^18^F]FE-PE2I PET. The striatum was segmented into ventral striatum, medial caudate, anterior putamen and posterior putamen. Mixed-effect models included region, hemisphere, and group as fixed effects, and subject ID as random effect. PD-MDD patients showed lower DAT binding in the ventral striatum (*P* = 0.016), but not in caudate or putamen, driving a significant group × region interaction (*P* = 0.012). Within the depressed group, greater depression severity was paradoxically associated with higher ventral striatal DAT binding (*P* = 0.039). These findings provide in vivo evidence linking ventral striatal dopaminergic dysfunction to PD-MDD, advancing mechanistic understanding relevant for treatment development.

## Introduction

Parkinson’s disease (PD) is the world’s fastest growing neurodegenerative disorder, with a current prevalence of almost 12 million patients worldwide, contributing to a growing public health challenge^1^. A common and debilitating non-motor symptom of PD is major depressive disorder (MDD), affecting approximately 35% of PD patients^2,3^. MDD in PD (PD-MDD) is associated with reduced quality of life^4^, increased motor symptom severity^5^ and mortality^6,7^. Despite its significant clinical impact, the pathophysiology of PD-MDD remains poorly understood. Dopaminergic dysfunction has been implied, but precise mechanisms remain unclear. This highlights the pressing need to better understand the role of dopamine in the pathophysiology of PD-MDD.

Alterations in dopamine signalling play an important role in MDD, also outside PD^8,9^. Dopamine regulates mood, psychomotor speed, decision-making, motivation and attention, all commonly affected in MDD. In PD, dopaminergic dysfunction is not restricted to the nigrostriatal pathway, which primarily contributes to motor symptoms. It also affects the mesolimbic and mesocortical pathways, associated with mood^10,11^. Pharmacological studies support this by showing the dopamine agonist pramipexole exerts antidepressant effects in PD-MDD^12,13^, and in MDD outside PD^14,15^, and that dopaminergic medication influences PD-MDD computational phenotypes^16,17^. Although other neurotransmitters systems have also been implicated in PD-MDD^18–20^, the dopaminergic system may play a key role. Investigating dopaminergic alterations in PD-MDD will clarify dopamine’s specific contribution to PD-MDD and improve our understanding of its pathophysiology.

Molecular imaging enables in vivo assessment of dopamine transporter (DAT) availability, reflecting presynaptic dopaminergic integrity. Previous DAT-imaging studies comparing PD patients with and without MDD have reported higher^21^, but generally lower striatal DAT availability^22–24^, though findings are inconsistent regarding region (caudate nucleus vs. putamen) and lateralisation. Notably, none of these studies specifically examined the ventral striatum, despite its key role in depression^25,26^. These inconsistencies may reflect methodological differences, including the variability in sample sizes across studies, which are often modest and sometimes uneven between comparison groups, as well as the limited spatial resolution and tracer selectivity of single-photon emission computed tomography (SPECT). SPECT ligands also bind to the serotonin transporter (SERT)^21–24^, obscuring how observed differences reflect dopaminergic alterations. Analyses based on left vs. right hemisphere may further obscure effects, given the asymmetric nature of PD. With [11C]RTI-32 positron emission tomography (PET), providing higher spatial resolution and better quantification than SPECT, Remy et al.^20^ showed reduced binding in PD-MDD compared to PD without MDD in several regions including the locus coeruleus, amygdala and ventral striatum. Despite better resolution, [11C]RTI-32 also binds to SERT and the norepinephrine transporter (NET), again preventing firm conclusions regarding DAT alterations.

Here, we innovatively use the highly selective [¹⁸F]FE-PE2I ((E)-N-(3-iodoprop-2-enyl)-2β-carbo[¹⁸F]-fluoroethoxy-3β-(4′-methyl-phenyl)nortropane) DAT tracer that has negligible affinity for SERT or NET, enabling an optimal assessment of DAT availability^27,28^ combined with high spatial resolution of PET. Furthermore, we also resolve other limitations of prior studies regarding the assessment of psychopathology: (i) instead of relying on (self-report) questionnaires of depression^21,23,24^, we ascertained MDD with a structured clinical interview by an experienced psychiatrist; (ii) we measured apathy and anhedonia to explain clinical heterogeneity in PD-MDD^29^, as dopamine is particularly implicated in anhedonic (and apathic) depression^30,31^.

This set-up enabled us to test the contribution of dopamine to PD-MDD with unprecedented rigour. We predicted that PD patients with MDD show reduced DAT binding in the striatum, particularly the ventral striatum (including the nucleus accumbens and ventral caudate nucleus), when compared with PD patients without MDD, and that this reduction is greater in patients with higher levels of anhedonia and apathy.

## Results

### Demographic and clinical characteristics

**Fig. 1:**
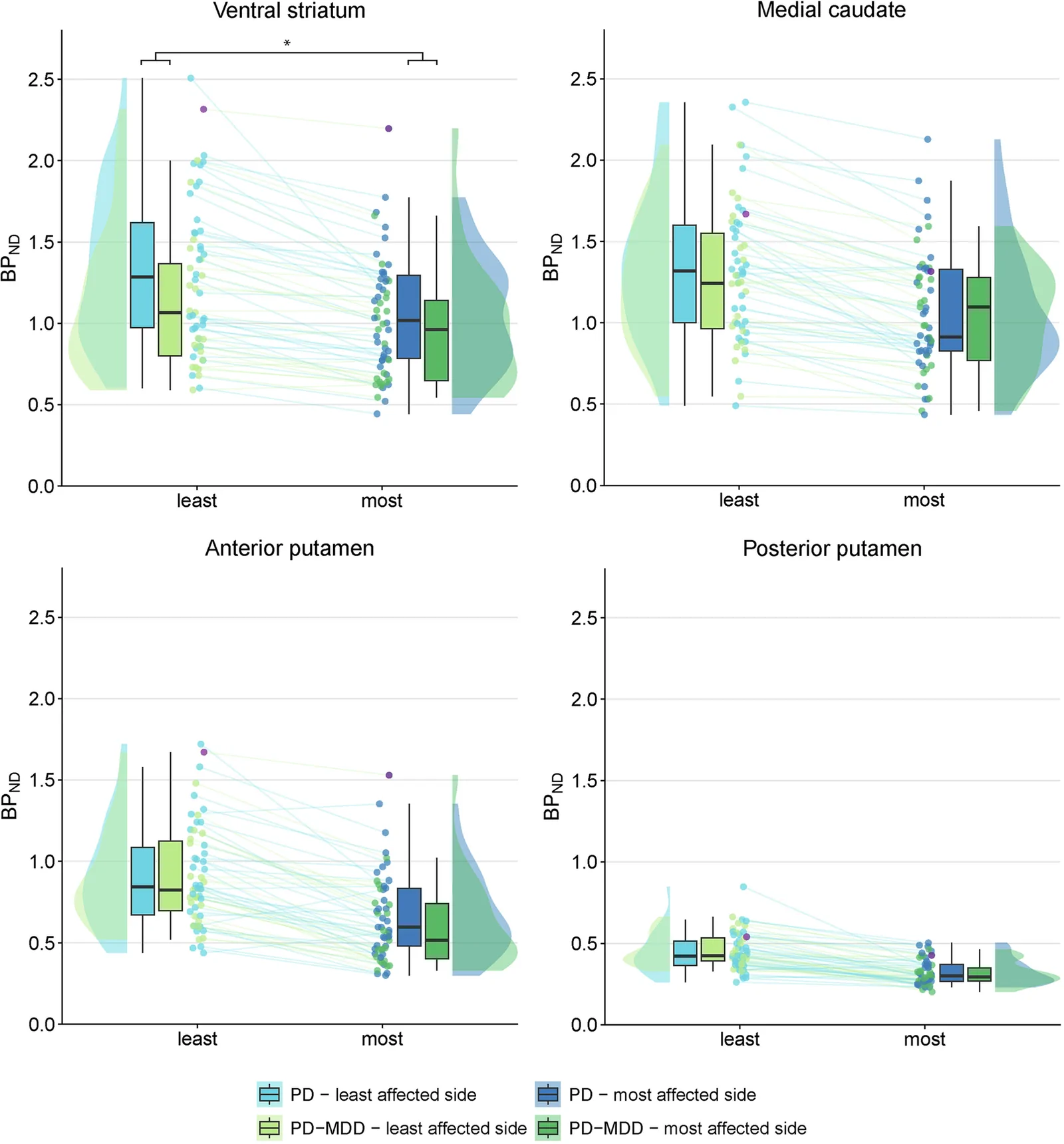
Striatal dopamine transporter (DAT) binding in depressed and non-depressed Parkinson’s disease (PD) patients. Violin plots, boxplots and individual data points of DAT non-displaceable binding potential (BP_ND_) values in the ventral striatum, medial caudate nucleus, anterior putamen and posterior putamen. Within each region, values are displayed separately for the most affected and least affected hemisphere (x-axis) and for depressed (*n* = 23, in green, the outlier not included in the analysis in purple) and non-depressed (*n* = 34, in blue) PD patients. Boxes indicate the interquartile range (IQR), horizontal lines the median and whiskers 1.5 × IQR. An asterisk indicates a significant group difference (*P* < 0.05) in the ventral striatum averaged over sides.

We recruited 28 depressed and 35 non-depressed PD patients. We excluded five from analysis due to a PET scan without evidence of dopaminergic deficit (SWEDD), and one because no MR data was available for co-registration. Moreover, following common outlier detection criteria (>3 SD from the mean), one participant was excluded from analyses (but not from display in Fig. 1, coloured in purple) due to extremely high BP_ND_ values in the anterior putamen and ventral striatum in the most affected hemisphere. Therefore 22 depressed and 34 non-depressed participants were included in the analyses (see demographic and clinical data in Table 1). Age, sex, Montreal Cognitive Assessment^32^ (MoCA) score, Movement Disorders Society-sponsored Revision of the Unified Parkinson’s Disease Rating Scale (MDS-UPDRS-III)^33^ scores and levodopa equivalent daily dose (LEDD) did not significantly differ between groups. Disease duration, however, was shorter in depressed compared with non-depressed patients (mean (SD): 3.75 (2.14) vs. 5.63 (1.98), *P* = 0.001). The proportion of dopamine agonist users did not significantly differ between groups, but the mean LEDD of dopamine agonists was lower in depressed than in non-depressed patients (27.81 (45.77) vs. 96.68 (131.13), *P* = 0.007). As expected, compared to the non-depressed group, the depressed group had significantly higher mean (SD) scores on depression (BDI-II: 26.82 (8.55) vs. 8.26 (6.65), *P* < 0.001), apathy (AS: 21.64 (5.10) vs. 11.15 (3.77), *P* < 0.001) and anhedonia (SHAPS: 32.05 (5.35) vs. 19.00 (4.66), *P* < 0.001; anticipatory-TEPS: 29.50 (9.70) vs. 39.82 (6.60), *P* < 0.001). Six depressed participants were using antidepressants at the time of PET scanning, of whom three used venlafaxine in doses ranging from 37.5 mg to 225 mg (allowed; non-dopaminergic). Two depressed participants were inadvertently scanned when using nortriptyline 30 mg and sertraline 50 mg (these drugs, by protocol, should have been discontinued prior to the PET scanning, which was overseen). In sensitivity-analyses, we post hoc repeated analyses excluding these two participants. Four additional participants had been using antidepressants (duloxetine, paroxetine, bupropion and nortriptyline) prior to PET scanning; however, these had been discontinued for at least five half-lives in accordance with the protocol.

**Table 1:** Demographic and clinical data from 22 depressed and 34 non-depressed PD patients

	Participants, Mean (SD)		
Characteristic	Depressed PD (*n* = 22)	Non-depressed PD (*n* = 34)	*P* value
Age	65.91 (6.03)	63.50 (8.39)	0.249
Sex ratio (male/female)	13/9	20/14	0.984
Disease duration (y)	3.75 (2.14)	5.63 (1.89)	0.001
MoCA^a^	27.00 (1.87)	26.56 (2.25)	0.455
MDS-UPDRS-III (OFF)^b^	34.57 (11.04)	34.00 (12.40)	0.864
MDS-UPDRS-Brad^b^	18.52 (7.12)	16.70 (6.78)	0.348
MDS-UPDRS-Rig^b^	5.81 (3.36)	6.42 (3.03)	0.489
MDS-UPDRS-Trem^b^	2.62 (2.64)	3.61 (3.65)	0.288
LEDD	590.88 (296.89)	700.62 (355.73)	0.235
DA-agonist use (y/n)	7/15	18/16	0.201
DA-agonist LEDD	27.81 (45.77)	96.68 (131.13)	0.007
BDI-II^c^	26.82 (8.54)	8.26 (6.65)	<0.001
Mild (14-19)	27%		
Moderate (20-28)	32%		
Severe (>28)	41%		
AS	21.64 (5.09)	11.15 (3.77)	<0.001
SHAPS	32.05 (5.35)	19.00 (4.66)	<0.001
TEPS (ant)	29.50 (9.70)	39.82 (6.69)	<0.001
Injected activity (MBq)	175.17 (10.10)	179.49 (9.41)	0.109

*MoCA* Montreal Cognitive Assessment, *MDS-UPDRS-III* Movement Disorders Society-sponsored Revision of the Unified Parkinson’s Disease Rating Scale part III, *MDS-UPDRS-Brad* bradykinesia subscore of the MDS-UPDRS (items 4-11 and 14), *MDS-UPDRS-Rig* rigidity subscore of the MDS-UPDRS (item 3), *MDS-UPDRS-Trem* resting tremor subscore of the MDS-UPDRS (items 17 and 18), *LEDD* levodopa equivalent daily dose, *DA* dopamine, *BDI-II* Beck Depression Inventory-II, *AS* Starkstein Apathy Scale, *SHAPS* Snaith Hamilton Pleasure Scale, *TEPS* Temporal Experience of Pleasure Scale (anticipatory items).

^a^Missing for 1 depressed PD patient who underwent a neuropsychological examination ruling out a diagnosis of dementia.

^b^Missing for 1 depressed and 1 non-depressed PD patient.

^c^Severity categories based on standard BDI-II cut-offs. Diagnosis of PD-MDD was based on clinical criteria, not BDI-II score.

### Lower ventral striatal DAT binding in depressed vs. non-depressed PD

The linear mixed-effects model revealed a significant main effect of region (*F*(3, 378) = 325.57, *P* < 0.001), indicating that BP_ND_ differed across at least two regions. It also showed overall lower BP_ND_ in the most vs. least affected hemisphere (main effect of side; *F*(1, 378) = 121.31, *P* < 0.001). In addition, there was a significant group × region interaction (*F*(3, 378) = 3.73, *P* = 0.012) (Fig. 1), which was confirmed by parametric bootstrapping (*n* = 1000 iterations). Post-hoc pairwise comparisons between groups for each region revealed significantly lower DAT binding in the ventral striatum of depressed vs. non-depressed PD patients with a large effect size (estimate = 0.187, SE = 0.076, *P* = 0.016, *d* = 0.90, 95% bootstrap CI [0.17, 1.63]), but not in the medial caudate nucleus (*P* = 0.32, *d* = 0.36, CI [−0.36, 1.09]), anterior putamen (*P* = 0.37, *d* = 0.33, CI [−0.39, 1.06]), or posterior putamen (*P* > 0.99, *d* = 0.00, CI [−0.73, 0.73]). The group × region interaction was robust: it remained significant after including preregistered covariates as fixed effects in the model (sex, age and MDS-UPDRS-III; *F*(3, 364) = 3.73, *P* = 0.012), and also after additionally including disease duration and dopamine agonist equivalent daily dose as covariates (*F*(3, 364) = 3.73, *P* = 0.012). Exploratory contrasts by side suggested that the group difference in the ventral striatum may be larger in the least affected (difference = 0.24, SE = 0.086, *t* = 2.80, *P* = 0.006) than in the most affected hemisphere (difference = 0.13, SE = 0.086, *t* = 1.56, *P* = 0.120). However, the full model did not show a significant group × side interaction, indicating these side-specific contrasts should be interpreted cautiously as descriptive observations. In another exploratory post hoc analyses, participants with depression preceding PD diagnosis (*n* = 11) showed numerically higher ventral striatal DAT binding than those with later-onset depression (*n* = 11), but differences were not statistically significant (least affected hemisphere: *P* = 0.306 one-sided; most affected hemisphere *P* = 0.282, one-sided). In post-hoc sensitivity analyses without the subjects with prohibited medication the primary linear mixed-effects model showed comparable results with a main effect of region (*F*(3, 364) = 303.92, *P* < 0.001), main effect of side (*F*(1, 364) = 111.39, *P* < 0.001) and a significant group × region interaction (*F*(3, 364) = 2.94, *P* = 0.033).

### Associations between ventral striatal DAT and clinical measures

**Fig. 2:**
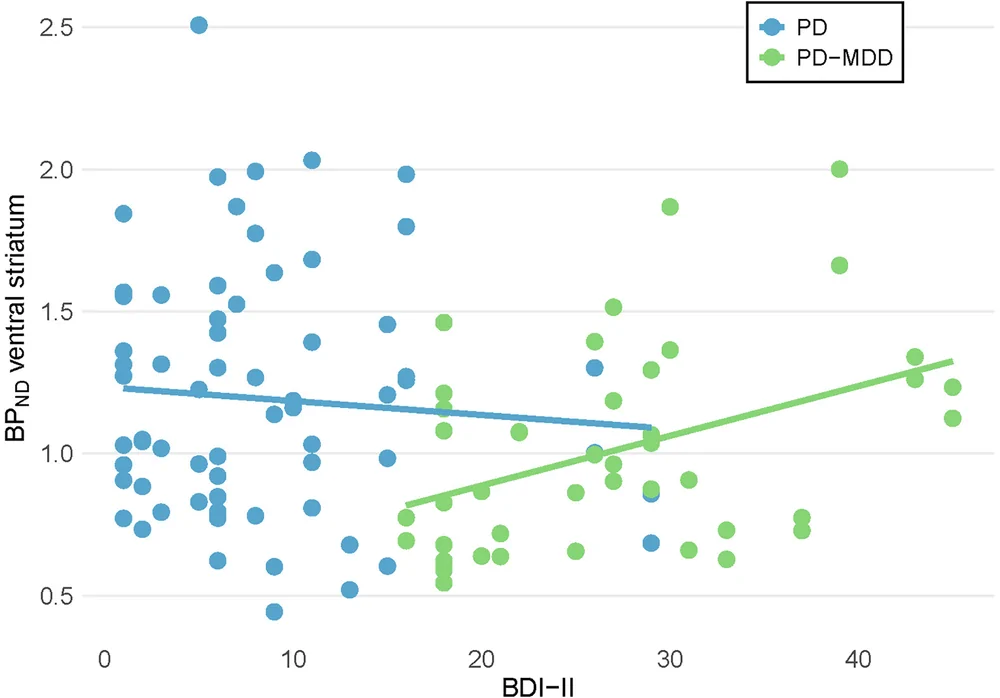
Association between ventral striatal dopamine transporter (DAT) availability and depression severity. Scatterplot of DAT non-displaceable binding potential (BP_ND_) values in the ventral striatum (y-axis) plotted against Beck Depression Inventory-II (BDI-II) scores (x-axis) for depressed (*n* *=* 22, in green) and nondepressed (*n* *=* 34, in blue) PD patients. Each patient contributes two points, one for each hemisphere. A fitted regression line is shown for illustration. Statistical associations were assessed using linear mixed-effects models. Post-hoc stratified analyses revealed a significant association between BDI-II scores and BP_ND_ in the depressed group (F(1, 20) *=* 4.90, *P* = 0.039), but not in the nondepressed group (F(1, 32) *=* 0.22, *P* = 0.640).

To investigate whether BP_ND_ in the ventral striatum correlates with the depression-severity, apathy, and anhedonia, linear mixed-effects models were fitted with sum scores of the BDI-II, AS, SHAPS and anticipatory-TEPS as fixed effects. For the BDI-II scores, there was a significant main effect of group (*F*(1, 52) = 4.66, *P* = 0.036; *β* = 0.42, 95% bootstrap CI [0.03, 0.81]), and a trend-level group × BDI-II interaction (*F*(1, 52) = 2.80, *P* = 0.100; *β* = −0.32, CI [−0.70, 0.06]) (Fig. 2). Post-hoc stratified analysis confirmed a significant positive association of BDI-II with BP_ND_ in the depressed group (*F*(1, 20) = 4.90, *P* = 0.039; β = 0.42, CI [0.04, 0.80]), reflecting a robust medium-sized effect, but not in the non-depressed group (*F*(1, 32) = 0.22, *P* = 0.640; *β* = −0.08, CI [−0.39, 0.24]). Because of the asymmetry between hemispheres we performed an additional exploratory linear mixed-effects model including side. This showed significant main effects of BDI-II and side, but no significant BDI-II × side interaction (*F*(1,20) = 0.03, *P* = 0.875), indicating the association between BDI-II and ventral striatal BP_ND_ in the depressed group did not differ between hemispheres. To exclude potential confounding by disease severity or duration, we performed additional moderation analyses within the depressed group. Neither MDS-UPDRS nor disease duration significantly moderated the association between BDI-II scores and ventral striatal BP_ND_ in the depressed group (*F*(1,17) = 0.41, *P* = 0.53 and *F*(1,18) = 0.25, *P* = 0.62, respectively). To account for potential effects of antidepressant use, exploratory analyses showed that antidepressant use was neither associated with BDI-II scores nor with ventral striatal BP_ND_ values, as users were distributed across the full range of symptom severity and DAT binding levels. Including AD use in the model did not alter the main effect of BDI-II and showed no main effect of AD use on BP_ND._ For AS, SHAPS, and anticipatory-TEPS scores, we found no significant main effects, nor interactions. Effect sizes for these measures were uniformly small (*β* = 0.02, 0.09 and 0.08, respectively), with wide CIs spanning zero.

## Discussion

In this first study using the highly selective DAT PET tracer [¹⁸F]FE-PE2I to investigate altered striatal dopamine function in PD patients with MDD versus non-depressed PD patients, we found that, in line with our hypothesis, depressed patients had lower DAT binding in the ventral striatum compared to non-depressed PD patients. Furthermore, contrary to our hypothesis, within the depressed group, ventral striatal DAT binding was positively associated with depression severity. These findings are consistent with a dopaminergic contribution to depression in PD.

Reduced ventral striatal DAT binding in PD-MDD aligns with a previous DAT PET study, although a nonselective tracer that also binds to NET was used^20^. Two SPECT studies found lower DAT in the caudate nucleus in PD-MDD^22,23^, but at lower spatial resolution and with anatomical definitions, so effects may have been driven by the head of the caudate nucleus, which is functionally part of the ventral striatum^34,35^. Moreover, substantial variability in sample sizes across these studies, ranging from very small (e.g. 12 vs. 8) to larger but uneven groups (e.g. 110 vs 30), likely contributes to the heterogeneity in reported findings and complicates interpretation. Outside PD, studies in MDD also reported lower ventral striatal DAT, though results were likely confounded by antidepressants^36^. Most findings in MDD indicated lower DAT in primarily putamen^37–39^. The absence of differences in putamen in our PD sample indicates that depressed and non-depressed PD groups were matched for dopaminergic denervation of the motor striatum, which is in line with the fact that motor symptoms were similar between groups. The positive association between ventral striatal DAT binding and depression severity in the depressed PD group, contrasts with previous studies of negative correlations in PD-MDD^23,24^. However, these previous studies employed tracers also binding to SERT, leaving uncertainty about whether effects were driven by serotonergic or dopaminergic effects. One study in healthy subjects, using a more DAT specific tracer (^99m^Tc-TRODAT-1), found a positive correlation between depression scores and DAT binding in the right caudate nucleus^40^, unfortunately the ventral striatum was not examined.

To interpret these findings, it is important to clarify that both degeneration of dopaminergic neurons and downregulation of DAT expression may reduce DAT binding, which PET cannot distinguish. We speculate in our PD sample the lower binding in the most affected hemisphere and posterior regions reflect neuronal degeneration. However, the ventral striatal DAT difference between depressed and non-depressed PD patients is less likely explained by degeneration alone, as the ventral striatum is relatively spared in early PD^41^, and the depressed group had a shorter disease duration. Post hoc analyses indicated the difference was more pronounced in the least affected hemisphere, where less degeneration has occurred, and more intact neurons allow regulation of DAT expression. Taken together, these findings suggest that downregulation of DAT may contribute to the observed group difference, potentially as an adaptive response to reduced dopamine signalling^9^. This is supported by imaging and postmortem findings in MDD outside of PD^37^. The positive association between ventral striatal DAT and depression could further support this hypothesis, but should be interpreted with caution, given the fact the association was only present in the depressed group with a relatively small sample size. It might suggest that more severely depressed patients exhibit a reduced capacity for compensatory downregulation, further lowering dopamine availability in the synaptic cleft. Similarly, a PET study by Meyer et al.^38^ in MDD reported lower overall striatal DAT in MDD, and showed higher DAT binding was associated with poorer dopamine-related neuropsychological performance. This is consistent with a reduced compensatory response, a phenomenon also suggested for SERT in seasonal affective disorder^42^. An additional hypothesis is that the neurobiological mechanisms underlying PD-MDD may differ depending on its temporal relationship to motor onset. Depression emerging after motor onset may be more closely related to dopaminergic degeneration, whereas pre-motor depression may reflect a greater contribution of the relatively reduced dopaminergic downregulation and/or involvement of non-dopaminergic neurotransmitter systems. However, post hoc stratification of the depressed group into pre- and post-motor onset subgroups did not reveal significant BP_ND_ differences. Given the limited sample size and exploratory nature of this analysis, this cannot be taken as evidence against neurobiological differences. Prospective studies with larger samples are needed to determine whether depression timing reflects distinct pathophysiological mechanisms.

Contrary to previous literature^29,43^ and our expectations, we found no correlation between DAT binding and self-reported apathy or anhedonia. Although limited power could be considered, the consistently small effect sizes and wide confidence intervals across all three measures suggest that the associations with ventral striatal DAT binding are genuinely small or absent, especially relative to the clearer effect in the BDI-II model. Importantly, several previous molecular imaging studies found group differences between depressed and non-depressed participants, but neither a correlation between striatal DAT binding and anhedonia scores^37,44^. Another PET-study reported no striatal DAT differences between anhedonic and non-anhedonic MDD-patients^45^. Evidence regarding apathy in PD is also mixed^46,47^, and while the Parkinson’s Progression Markers Initiative cohort showed a correlation between striatal DAT and an apathy and anhedonia composite score, this only emerged at follow-up, after PD progressed^48^. To accommodate that the SHAPS primarily assesses consummatory pleasure, whereas dopamine is more closely associated to anticipatory anhedonia, we additionally used the anticipatory items from the TEPS. However, we found no significant correlation with DAT binding. Since the motivational ‘wanting’ aspect of anhedonia is difficult to capture with self-report questionnaires, exploration of this implicit behavioural dimension might better be quantified with a behavioural task^49^.

The present findings may help refine treatment strategies for PD-MDD, suggesting that interventions targeting dopaminergic pathways could address its underlying mechanisms. Although LEDD did not differ significantly between groups, a positive correlation between LEDD and BDI-II scores in the depressed group (*ρ* = 0.42, *P* = 0.049) may indicate that dopaminergic therapy had already been escalated in an attempt to manage mood symptoms. Notably, dopaminergic agonist use was more common in the non-depressed group, who also had a longer disease duration. While it is conceivable to relate these factors to one another, caution is warranted given the complex aetiology of PD-MDD and the difficulty of causal inferences from our cross-sectional design. Mechanistically, if impaired DAT downregulation limits synaptic dopamine availability, increasing levodopa alone may be insufficient to normalise dopaminergic tone, whereas agonists can partially bypass this limitation by acting postsynaptically. These considerations suggest that strategies directly targeting DAT, such as methylphenidate or modafinil, or enhancing postsynaptic dopaminergic signalling, may be more rational than further levodopa escalation. Methylphenidate has demonstrated antidepressant efficacy in PD-MDD^50^, and late-life depression^51^, yet remains underutilized, whereas modafinil, which has shown modest antidepressant effects as monotherapy or augmentation in MDD^52,53^, has primarily been studied in PD for fatigue rather than mood^54^. Overall, these observations highlight the need to for further investigation of DAT-targeted pharmacological strategies as potential treatments for PD-MDD.

A major strength of this study is the use of the highly selective [¹⁸F]FE-PE2I DAT tracer, that has negligible binding to SERT and NET, ensuring that the measured signal reflects dopaminergic processes^27,28^. Furthermore, T1-weighted MRI scans enabled precise delineation of regions of interest (ROIs). Together, these factors enabled the highest-possible resolution PET imaging of the DAT system in PD patients in a large sample. Furthermore, we utilised a validated clinical diagnosis of MDD to classify participants as depressed. This is crucial given the high prevalence of depressive symptoms and their overlap with PD symptoms, even when clinical depression is not present^55,56^. A first limitation is that, although groups were matched for age, sex and MDS-UPDRS-III scores, they differed in disease duration. The depressed group had a shorter disease duration, excluding the possibility that reduced DAT binding reflected disease progression over time. This difference may even have led to an underestimation of the group effect. Variability in diagnostic delay could partly explain this discrepancy. Although the effect size for disease duration was large (Cohen’s *d* = 0.93), the clinical relevance of this 2-year difference is likely limited. Importantly, our study focuses on depression in relatively early-stage PD (mean disease duration 3.75 years, range 0.25–9.25), and mechanisms underlying depression at later stages may differ and are not captured in this cohort. Second, participants were scanned while on dopaminergic medication. Although such medication is not expected to substantially affect DAT binding, minor effects cannot be ruled out^57,58^. However, any influence would likely affect the striatum uniformly rather than the ventral striatum specifically^59^. In addition, although we excluded antidepressants with evident DAT binding and restricted antidepressant dosage, it could still be argued that antidepressants may influence DAT availability^60^ and thus confound the observed association between BDI-II score and BP_ND_. In our sample, however, antidepressant use was not associated with BDI-II scores or BP_ND_ values, and adjusting for antidepressant use did not alter the results, suggesting that our findings were unlikely to be driven by antidepressant use. Moreover, from a clinical and ethical perspective, discontinuation of antidepressants is often not feasible in patients with MDD due to potential risks associated with treatment interruption. Third, cerebrovascular pathology was not systematically assessed using MRI sequences sensitive to small-vessel disease. Although screening of medical history did not suggest prevalent cerebrovascular disease in the cohort, subclinical vascular changes cannot be fully excluded. Finally, the cross-sectional design precludes causal conclusions. Longitudinal studies are required to establish the temporal relationship between DAT alterations and the occurrence of PD-MDD.

In conclusion, our findings provide evidence for a dopaminergic contribution to depression in PD and localises the dopaminergic deficit specifically to the ventral striatum. This may be clinically relevant by supporting the use of dopaminergic medication to treat not only motor symptoms but also depressive symptoms in PD. Future longitudinal studies are needed to clarify the causal relationship between DAT alterations and PD-MDD, and to test the efficacy of DAT-targeted pharmacological strategies as potential treatments.

## Methods

### Participants and design

After approval by the central Committee on Research Involving Human Subjects (CCMO, #NL8664/CMO2020-6619) and the local Medical Ethics Committee (METC Oost-Nederland), and written informed consent in accordance with the Declaration of Helsinki, we obtained data from depressed and non-depressed PD patients in the cross-sectional AnHedonic dEpression and Alterations in the Dopaminergic neurocircuitry in Parkinson’s disease (AHEAD) study. Patients were recruited via the Personalized Parkinson’s Project^61^ and outpatient clinics of the Radboudumc and affiliated hospitals. All had PD diagnosed within the past 10 years, confirmed by experienced neurologists according to established international diagnostic criteria^62^, and were currently using dopaminergic medication. Exclusion criteria included contraindications for magnetic resonance (MR) or PET imaging (claustrophobia, epilepsy, inability to lie flat or lie still for the duration of the scan, presence of an active implant (e.g. pacemaker, insulin pump, neurostimulator, ossicular prostheses), and/or other medical device or other non-removable metal part), dementia (MoCA score < 21/30), bipolar disorder, current psychotic symptoms, or the inability to discontinue drugs or medication with evident DAT binding. Excluded was use of methylphenidate, bupropion, amphetamines, or cocaine. We did allow use of antidepressants, with the exception of those antidepressants with a high DAT binding defined as a relatively low Ki of <1000: bupropion, duloxetine, and sertraline. Moreover, we did not include patients using antidepressants at higher than minimal effective dosages used for antidepressive effects when the Ki is <10,000. The maximum dosage allowed was: amitriptyline 25 mg, clomipramine 25 mg, maprotiline 25 mg, nortriptyline 25 mg, fluoxetine 10 mg, paroxetine 10 mg. All eligible participants underwent a structured clinical interview for DSM-5 (SCID-5-S)^63^. Based on this interview, participants were included in either the depressed or non-depressed group. Non-depressed participants did not meet the DSM criteria for MDD at the time of inclusion and had not met criteria for MDD within the preceding 5 years, as assessed during the SCID-5-S. Groups were matched for age, sex and disease severity, measured by part III of the MDS-UPDRS in off-medication state.

### Measurements

Participants completed several questionnaires assessing psychiatric symptoms: the Beck Depression Inventory-II (BDI-II)^64^ was used for depression severity, and the Starkstein Apathy Scale (AS)^65^ for apathy, both validated in PD^66,67^. The Snaith Hamilton Pleasure Scale (SHAPS)^68^ was used for anhedonia and is also validated in PD^67^. Because the SHAPS primarily focusses on the consummatory aspect of anhedonia, we also included the anticipatory items of the Temporal Experience of Pleasure Scale (TEPS)^69^ to capture the motivational aspect of anhedonia. Motor symptoms were assessed using the MDS-UPDRS-III^33^, subscores were calculated as described by Zach et al.^70^, and levodopa equivalent daily dose (LEDD) was calculated as described before^71^. MR and dynamic PET data were then acquired. Since PD medication is not expected to influence DAT binding or imaging outcomes during PET scanning^57,58^, all measurements (except MDS-UPDRS-III) were obtained in the on-medication state.

### Imaging data acquisition and reconstruction

Dynamic PET data were acquired using Biograph mCT PET/CT scanner (Siemens Healthineers, Erlangen, Germany) at the department of Medical Imaging, Radboudumc, Nijmegen. A head holder was employed to minimise head movements during scanning. For attenuation correction, a low-dose CT scan was performed (voxel size 3.0 × 3.0 mm; pitch 1.0; 16 × 1.2 mm), with automatically modulated X-ray tube voltage (120 kV) and current (50 mA) using Care kV and Care Dose4D (Siemens Healthcare). The radiotracer [^18^F]FE-PE2I was produced by Radboud Translational Medicine B.V. (Nijmegen, the Netherlands) and was synthesised in accordance with the Good Manufacturing Practice and current norms of radioprotection. All participants received a bolus injection of approximately 185 MBq [^18^F]-FE-PE2I intravenously in 10 s, after which the syringe was flushed with 40 mL Nacl 0.9%. The syringe was measured for residual activity to obtain the total injected dose. The PET data were acquired in list-mode over 90 min and reconstructed in 36 frames (6 × 10 s, 6 × 20 s, 4 × 30 s, 4×60 sec, 5 × 180 s, 11 × 360 s). Images were reconstructed with the TrueX algorithm incorporating time-of-flight information (3 iterations, 21 subsets, a 400 × 400 matrix) to a voxel size of 1.02 × 1.02 × 3.00 mm^3^, with a Gaussian post-reconstruction filter of 3.0 mm in full-width at half-maximum (FWHM).

MR images of the brain were obtained for co-registration to the PET data using a Siemens MAGNETOM PrismaFit 3 T MRI scanner with a 32-channel head coil (Siemens Healthineers, Erlangen, Germany). High resolution structural data were acquired using a T1-weighted magnetisation-prepared rapid acquisition with gradient multi echo (MPRAGE) sequence (Time (TR) = 2000 ms; echo time 2.0 ms; flip angle 8 degrees; field of view = 256 mm; voxel size 1 × 1 × 1 mm^3^). One patient with a residual dental metal fragment was, due to safety considerations, scanned at the Radboudumc hospital, with a similar Siemens PrismaFit 3T MRI scanner with a 32-chennel head coil, and a slightly different but comparable sequence (TR = 2300 ms; echo time 2.32 ms; flip angle 8 degrees; field of view 240 mm; voxel size 0.9 × 0.9 × 0.9 mm^3^).

### Preprocessing

Preprocessing of the PET data was performed using the PNEURO module of PMOD (version 4.405; PMOD Technologies Ltd., Zurich, Switzerland). The PET data were smoothed with a Gaussian kernel of 6.0 mm FWHM before applying frame-by-frame movement correction to frames 7–36 with the average of frames 7–15 as reference. The MR images were parcellated and subsequently matched to the subject’s PET series within the brain parcellation workflow^72^. Outlining of the cerebellum was visually inspected and manually corrected if necessary. Partial volume correction (PVC) was then applied^73^, using a simplified Müller-Gartner approach^74^, to account for spill-out of PET signal. Following PVC, the time activity curves (TAC) were extracted for the striatum as a whole and the cerebellum.

### ROI analysis

**Fig. 3:**
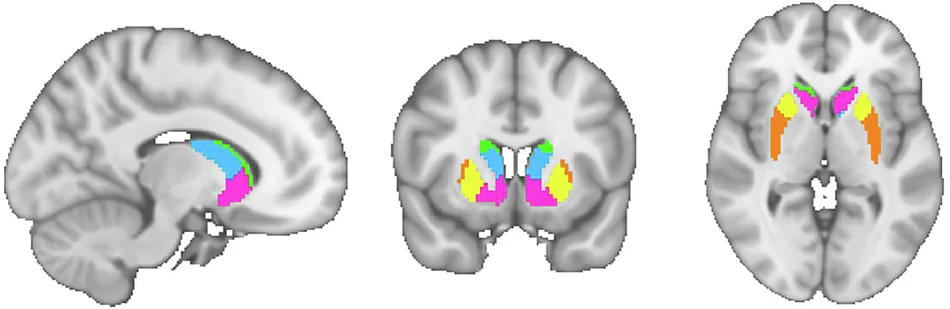
Functional connectivity based parcellation of the striatum. The parcellation consists of the ventral striatum (including nucleus accumbens, ventral caudate nucleus and most ventral parts of the putamen; in magenta), medial caudate nucleus (in blue), dorsal caudate nucleus (in green), anterior putamen (in yellow) and posterior putamen (in orange)^34^.

ROIs were then defined using a striatal parcellation based on k-means clustering of functional connectivity, which more accurately reflects function than anatomical divisions^34^, and included left and right ventral striatum (including nucleus accumbens, ventral caudate nucleus, and most ventral parts of the putamen), medial caudate nucleus, anterior putamen and posterior putamen (Fig. 3). The dorsal caudate nucleus was excluded due to its small volume and close proximity to the ventricles, which complicates reliable PET signal extraction, particularly in older brains^73,75^. MR images were first brain-extracted using FSL version 6.0.6^76,77^. To derive transformations between PET, MR and MNI152 space, FLIRT/FNIRT were applied^78,79^, enabling projection of the functional subdivision onto native PET space. Grey matter of the cerebellum served as the reference region due to its negligible DAT density^80,81^. The outlined ROIs on the dynamic PET scan were combined with TACs from the cerebellum and the striatum as a whole (for k₂′ estimation) to compute DAT non-displaceable binding potential (BP_ND_) using the simplified reference tissue model (SRTM)^81,82^ implemented in MATLAB (R2024b).

### Statistical analysis

Statistical analyses were performed in RStudio 2024^83^, following a preregistered plan (https://doi.org/10.17605/OSF.IO/4UFPB). Group differences in age, disease duration, MoCA score, MDS-UPDRS-III, LEDD, DA-agonist LEDD, BDI-II, AS, SHAPS, anticipatory-TEPS and injected radioactivity were assessed with independent *t*-tests; sex and dopamine agonist use were tested with chi-squared. Linear mixed-effects models (lmer) were used with BP_ND_ as the dependent variable, region (ventral striatum, medial caudate nucleus, anterior putamen, posterior putamen), side (most and least affected hemisphere, based on MDS-UPDRS-III) and group (depressed vs. non-depressed) as fixed effects, and subject ID as random effect (BP_ND_ ~ group * region * side + (1 | Subject ID), using Type III ANOVA (Satterthwaite). Separate models included preregistered covariates age, sex and MDS-UPDRS-III as fixed effects. The associations of the clinical measures (BDI-II, AS, SHAPS, anticipatory-TEPS) with BP_ND_ were investigated using mixed-effects models including continuous scores and group as fixed effects. Post hoc pairwise comparisons of estimated marginal means were conducted using the emmeans package in R. To address concerns regarding statistical power, parametric bootstrap methods (*n* = 1000 iterations) were applied to all models using the bootMer function in the lme4 package in R. Effect sizes were calculated for all comparisons: Cohen’s *d* for the post-hoc pairwise comparisons of the primary model, and standardised coefficients (*β*) for the mixed-effects models examining associations with clinical scales.

To exclude potential confounding by disease severity or duration were performed additional moderation analyses within the depressed group with the BP_ND_ in the ventral striatum as dependent variable, the mean centred BDI-II score and MDS-UPDRS-III score or disease duration as fixed effects and subject ID as random effect (BP_ND_ ~ BDI-II * MDS-UPDRS-III + (1 | Subject); BP_ND_ ~ BDI-II * disease duration + (1 | Subject), using type III ANOVA with Satterthwaite). To rule out antidepressant use as confounder, we included antidepressant use during the PET scan in the model (BP_ND_ ~ BDI-II + AD use + (1 | Subject), using type III ANOVA with Satterthwaite). Additional exploratory analyses were performed to further explore the results, as detailed in the ‘Results’ section.

## Data Availability

The data are not publicly available due to privacy concerns. Pseudonymized and encrypted data supporting the findings of this study are available from the corresponding author upon reasonable request, and subject to institutional approval in line with participant consent and ethical approval.
